# Association Between Catalase Gene Polymorphisms and Risk of Chronic Hepatitis B, Hepatitis B Virus-Related Liver Cirrhosis and Hepatocellular Carcinoma in Guangxi Population: A Case–Control Study

**DOI:** 10.1097/MD.0000000000000702

**Published:** 2015-04-03

**Authors:** Yanqiong Liu, Li Xie, Jiangyang Zhao, Xiuli Huang, Liuying Song, Jingrong Luo, Liping Ma, Shan Li, Xue Qin

**Affiliations:** From the Department of Clinical Laboratory, First Affiliated Hospital of Guangxi Medical University, Nanning, Guangxi, China.

## Abstract

Reactive oxygen species (ROS) play critical roles in hepatocarcinogenesis. The catalase (CAT) enzyme is involved in the repair of ROS. Therefore, we investigate the association between *CAT* gene polymorphisms and the risk of hepatocellular carcinoma (HCC).

A total of 715 subjects were divided into 4 groups: 111 chronic hepatitis B (CHB) patients, 90 hepatitis B virus (HBV)-related liver cirrhosis (LC) patients, 266 HBV-HCC patients, and 248 healthy controls. The polymerase chain reaction-restriction fragment length polymorphism strategy was used to detect CAT gene rs1001179, rs769217, and rs7943316 polymorphisms.

Binary logistic regression analyses adjusting for sex, age, ethnicity, smoking and alcohol consumption, and body mass index suggested that subjects carrying the rs769217 T allele were at marginally increased risk of CHB, LC, and HCC, with adjusted odds ratios (ORs) of 1.51 (95% confidence interval [CI] = 1.04–2.20, *P* = 0.029), 1.48 (95% CI = 1.03–2.14, *P* = 0.035), and 1.51 (95% CI = 1.14–1.98, *P* = 0.004), respectively. Similarly, those individuals carrying the rs769217 TT genotype had a moderately increased risk of CHB, LC, and HCC, with adjusted ORs of 2.11 (95% CI = 1.05–4.22, *P* = 0.035), 2.00 (95% CI, 1.01–3.95, *P* = 0.047), and 1.93 (95% CI = 1.14–3.28, *P* = 0.015), respectively. Moreover, subjects carrying the rs769217 CT genotype and at least 1 copy of the T allele (dominant model) were 1.78 times and 1.83 times more likely to develop HCC, respectively (OR = 1.78, 95% CI = 1.16–2.73, *P* = 0.009 and OR = 1.83, 95% CI = 1.23–2.71, *P* = 0.003). This association between CAT rs769217 T alleles and HCC risk is significantly strengthened among men, nonsmokers, nondrinkers, and among individuals <50 years of age. Furthermore, we found 1 high-risk haplotype GTA for CHB (OR = 1.45, 95% CI = 1.05–2.01) and 1 protective haplotype GCA for HCC risk (OR = 0.67, 95% CI = 0.52–0.87). We did not found any significant difference in CAT rs1001179 and rs7943316 polymorphisms between controls and cases.

Our findings suggest that the CAT rs769217 T allele is associated with increased risk of CHB, HBV-LC, and HBV-HCC in Guangxi population.

## Abstract 2

Supplemental Digital Content is available in the text

## INTRODUCTION

Hepatocellular carcinoma (HCC) is one of the most frequently occurring cancers and a leading cause of cancer-related deaths worldwide.^1^ According to the latest report from the American Cancer Society, in 2015, 35,660 new liver cancer cases will be diagnosed, and 24 550 estimated deaths from liver cancer are predicted in the United States.^1^ The distribution of HCC varies widely in different geographic regions worldwide, and China alone accounts for an estimated half of all HCC cases.^2^ The carcinogenesis of HCC is a multifocal and complex process, and the etiology remains largely elusive. Currently, the well-recognized risk factors for HCC include chronic viral hepatitis, smoking, alcohol consumption, aflatoxin exposure, and liver cirrhosis.^3–5^ However, only a fraction of people with established risk factors eventually develop HCC, suggesting that other genetic and environmental mediators may be involved in HCC development.

It has been suggested that oxidative stress plays a critical role in the initiation and progression of hepatocarcinogenesis.^6,7^ Oxidative stress can cause an imbalanced antioxidant defense system or excessive reactive oxygen species (ROS) production.^8^ ROS can cause severe DNA damage.^8,9^ Catalase (CAT) is an endogenous antioxidant enzyme involved in ROS neutralizing pathways that in turn are involved in mechanisms against oxidative stress.^10^ CAT can catalyze the conversion of hydrogen peroxide (H_2_O_2_) (a type of ROS), to water (H_2_O) and oxygen (O_2_), thereby preventing cell injury from ROS.^10^ Allelic variants in the antioxidant genes coding for CAT enzymes may have deleterious effects on the expression or function of CAT, which may result in lower CAT enzymatic activity and higher sensitivity to ROS.^10,11^ Therefore, genetic variations in CAT may alter ROS detoxification and increase oxidative stress, implicating oxidative DNA damage and modulating disease risk.^11^

The human *CAT* gene, encoded by the nuclear chromosome 11p13, consists of 13 exons and 12 introns.^12^ A series of single-nucleotide polymorphisms (SNPs) in the *CAT* gene have been identified.^13,14^ Genetic polymorphisms located in the promoter region could influence rates of transcription, resulting in low CAT activity.^13,15^ The most studied mutation is the rs1001179 SNP, which is located in the 5′-UTR, the 262 base pairs from the transcription start site of the *CAT* gene.^16^ The variant A allele of the CAT rs1001179 polymorphism was associated with lower CAT enzyme activity compared with the G allele, and thus increased levels of ROS.^16^ Besides this SNP, rs769217 was a T/C silent substitution in CAT codon 389 of exon 9. Although it is a silent substitution, it contains hidden changes that constitute informative gene markers for the *CAT* gene.^17^ Another common polymorphism in the promoter region of the *CAT* gene consists of A to T at exon 2, codon 21.^18^ CAT rs7943316, which is situated inside the promoter region just proximal to the start site, may influence the gene expression by its position close to the start codon or linkage to the interest areas of the promoter where transcription factors are tied up.^19^

Several research have investigated the association between the CAT SNPs rs1001179, rs769217, and rs7943316 polymorphisms and the risk of various cancers such as breast cancer,^20^ cervical cancer,^21^ prostate cancer,^22^ pancreatic cancer,^23^ and colorectal cancer^24^ in various races. Three studies to date have examined the influence of CAT polymorphism on the risk of HCC.^25,26^ One study, assessing 96 Moroccan patients with HCC, reported that male patients carrying the CAT rs1001179 TT genotype had a significantly higher risk of developing HCC compared with controls (odds ratio [OR] = 15.94, 95% CI = 3.48–72.92, *P* < 0.001).^26^ One study, assessing 190 French patients with HCC, found no overall association with risk.^25^ Another study, assessing 106 Korean patients with HCC, also found nonsignificant association between *CAT* gene polymorphisms and HCC risk.^27^ However, the sample sizes of these 3 studies were very limited, and only 1 SNP (rs1001179 or rs7943316) of the *CAT* gene was investigated. In addition, the genotype distributions of the CAT polymorphisms vary with ethnicity, and until now no study has been carried out on the association between the CAT polymorphisms and HCC risk in the Chinese population. Therefore, we further evaluate the association between these 3 widely studied *CAT* gene polymorphisms (rs1001179, rs769217, and rs7943316) and the risk of hepatitis B virus (HBV)-related HCC in addition to chronic hepatitis B (CHB) and HBV-liver cirrhosis (LC) in the Guangxi Chinese population.

## MATERIALS AND METHODS

### Study Population

This was a retrospective, case–control study approved by the ethics committee of the First Affiliated Hospital of Guangxi Medical University, Guangxi, China. All of the involved patients and all healthy volunteers provided written informed consent. The cases were consecutively recruited from the patients who were evaluated and treated at the First Affiliated Hospital of Guangxi Medical University from April through October of 2014.

The inclusion criteria are listed further and were also described in detail previously.^28–30^ Patients were all confirmed to have had a history of HBV infection for at least 6 months. CHB was defined as serum HBV-DNA levels of ≥1000 copies/mL and elevated alanine aminotransferase (ALT) or aspartate aminotransferase (AST) (>40 IU/mL). A total of 111 patients with CHB fulfilled the selection criteria and were successfully genotyped. HBV-LC was diagnosed based on the combination of clinical history, pathologic examination, imaging and laboratory data, and/or histology. At last, 90 HBV-LC fulfilled the selection criteria and were successfully genotyped. HBV-HCC was diagnosed based on either histological or cytological findings or on elevated serum alpha fetoprotein (AFP) levels (>400 ng/mL) combined with at least 1 positive liver image on computed tomography, magnetic resonance imaging, or ultrasonography. In our study, 266 HCC cases fulfilled the selection criteria and were successfully genotyped.

Patients were excluded if they had any of the following conditions: other concomitant causes of liver disease or mixed etiologies (hepatitis A/C/D/E virus, autoimmune hepatitis, primary biliary cirrhosis, alcoholic hepatitis); had a family history of HCC or other concomitant malignant neoplasias; or had a history of autoimmune or inflammatory diseases such as systemic lupus erythematosus, diabetes mellitus, rheumatoid arthritis, or inflammatory bowel disease.

Controls who were negative for HBV markers and without any clinical evidence of hepatic disease or tumor were randomly recruited from a pool of healthy volunteers who visited the general health check-up centers at the same hospitals during the same time frame. In this study, 248 healthy controls fulfilled the selection criteria and were successfully genotyped.

Demographic and laboratory data were obtained using electronic medical records. Age, sex, history of smoking and alcohol use, ethnicity, and body mass index (BMI) were analyzed. Separated serum samples were tested for HBV and HCV markers, AFP levels, serum total bilirubin (TBIL), total protein (TP), albumin (ALB), gamma glutamyl transpeptidase (GGT), AST, and ALT. An alcohol drinker was defined as someone who consumed alcoholic beverages at least once per week for more than 6 months. Subjects were considered tobacco smokers if they smoked up to 1 year before the date of diagnosis for cases, or up to the date of interview for controls.

### Sample Size Consideration

We estimated the sample size using Quanto software (version 1.2.4) based on probability of α = 0.05 and β = 0.1 and assuming that the prevalence of the rs769217 CC genotype in the control group was 20.9% (HapMap Project dbSNP database: http://www.ncbi.nlm.nih.gov/snp/), and estimated OR was 2.0. The inheritance model was recessive. Approximately 1 to 1 control–case ratio was chosen. According to the parameters mentioned earlier, the estimated 157 sample size had enough power to assess the risk of the *CAT* genetic variation on HCC development.

### DNA Extraction

Peripheral blood samples (2 mL) were collected from all of the subjects in ethylenediaminetetraacetic acid-coated vials and stored at −20°C until DNA extraction. Genomic DNA was isolated from peripheral leukocytes using the phenol-chloroform extraction method. The blood samples were submitted to digestion in sodium dodecyl sulfonate, followed by phenol extraction twice and chloroform extraction once. DNA was then ethanol-precipitated and resuspended in buffer solution DNA concentration was determined spectrophotometrically.

### Genotyping of the *CAT* Gene

Genotyping of CAT SNPs rs1001179, rs769217, and rs7943316 was performed by polymerase chain reaction (PCR) restriction fragment length polymorphism. The PCR was carried out in a final volume of 25 μL, consisting of 2 μL of genomic DNA, 1 μL of each primer, 12.5 μL of Green PCR Master Mix (Shanghai Sangon Biotech Co., Ltd., Shanghai, China), and 8.5 μL of nuclease-free water. For rs1001179, rs769217, and rs7943316, 10 μL aliquots of the PCR products were digested in 1 μL of *Sma*I, *Bst*XI, or *Hinf*I restriction enzymes, respectively. The primers used for amplification and the cycling conditions are listed in Table 1.

**TABLE 1:**
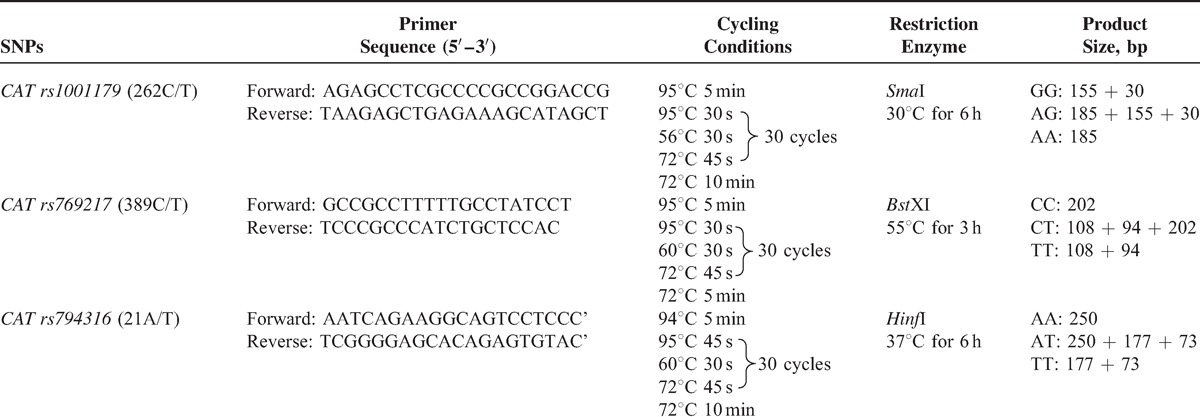
Primer Sequence and the Reaction Condition for Genotyping CAT Polymorphisms

**FIGURE 1:**
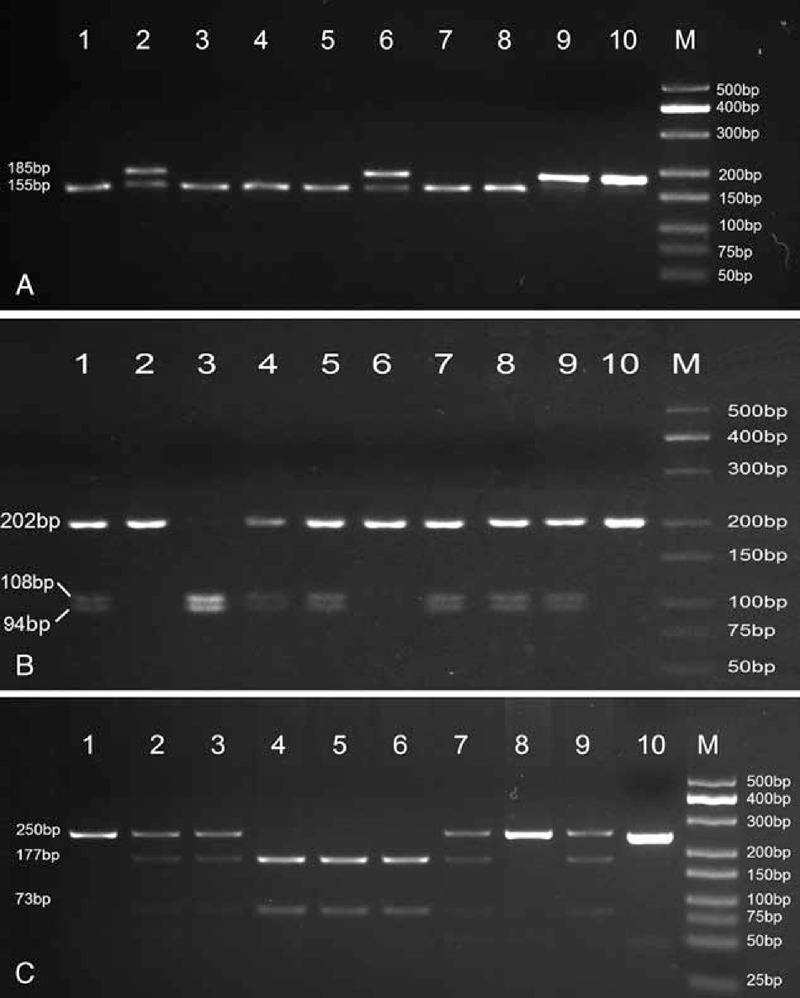
PCR-RFLP assay for analyzing the rs1001179, rs769217, and rs7943316 polymorphisms in *CAT* gene. (A) rs1001179—lanes M: DNA marker; lanes 1, 3, 4, 5, 7, and 8 show GG genotype; lanes 2 and 6 show AG genotype; lane 9 shows AA genotype; lane 10 shows negative control. (B) rs769217—lanes M: DNA marker; lanes 1, 4, 5, 7, 8, and 9 show CT genotype; lanes 2 and 6 show CC genotype; lane 3 shows TT genotypes; lanes 10 shows negative control. (C) rs7943316—lanes M: DNA marker; lanes 1 and 8 show AA genotype; lanes 2, 3, 7, and 9 show AT genotype; lane 4, 5, and 6 shows TT genotypes; lanes 10 shows negative control. PCR-RFLP = polymerase chain reaction-restriction fragment length polymorphism.

Digested fragments were separated by electrophoresis in 2% agarose gel containing GoldView I (Beijing Solarbio Science & Technology Co., Ltd., Beijing, China) and the fragments were visualized by the UV transilluminator (Figure 1). To control the quality of genotyping, a negative control was performed in each genotyping assay. The negative control utilized a PCR-amplified DNA product without the restriction enzymes.

In addition, a total of 70 specimens (about 10%) were randomly selected and genotyped by DNA sequencing with an ABI Prism 3100 (Applied Biosystems, Shanghai Sangon Biological Engineering Technology & Services Co., Ltd., Shanghai, China). The results of DNA sequencing were 100% concordant.

### Statistical Analysis

The distribution of the general demographic and clinical features between cases and controls was evaluated by using the 1-way analysis of variance test and the χ^2^ test for continuous and categorical variables, respectively. Agreement with Hardy–Weinberg equilibrium (HWE) for each SNP was tested using a goodness-of-fit χ^2^ test. Genotype frequencies were compared among different groups using the χ^2^ test and Fisher exact test when appropriate. The haplotype analyses were performed using SHEsis software (http://analysis.bio-x.cn/myAnalysis.php).^31^ A binary logistic regression model was used to obtain the estimated ORs and 95% confidence intervals (CIs) after adjusting for potential confounding variables such as sex, age, ethnicity, smoking and alcohol consumption, and BMI. To investigate the effect of other potential confounding variables on the association between *CAT* genetic variants and HCC risk, we also stratified our population according to sex, age, history of smoking, and alcohol consumption. A 2-tailed *P* value of <0.05 was considered statistically significant. All of the statistical analyses were performed in SPSS version 13.0 software (SPSS Inc., Chicago, IL).

## RESULTS

### Characteristics of the Study Population

The demographic and clinical characteristics of the controls and cases are shown in Table 2. The mean ages (±standard deviation) of the control group, CHB, LC, and HCC groups were 46.50 ± 6.94, 38.14 ± 11.88, 49.83 ± 12.11, and 49.38 ± 11.14 years, respectively. The CHB patients were significantly younger than the control group, LC, and HCC patients. Patients with HBV infection were more likely to be male (*P* < 0.001), smoke more tobacco (*P* = 0.036), and drink more alcohol (*P* < 0.001). The values of AFP, TBIL, AST, and ALT were significantly higher in HBV-infected patients than in control subjects (*P* < 0.001). There were no significant differences for ethnicity, BMI, TP, ALB, and GGT between these 4 groups.

**TABLE 2:**
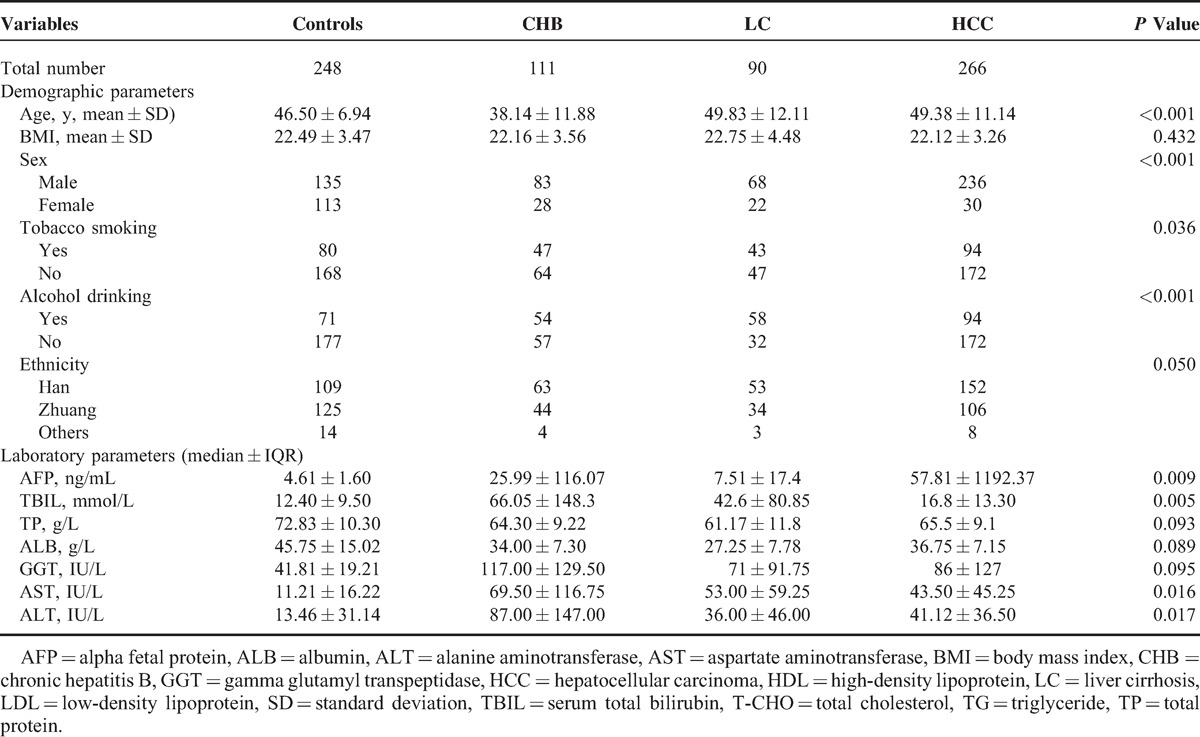
Baseline Characteristics of the Study Population

### Alleles and Genotype Distributions of CAT Polymorphisms in Healthy Controls, CHB, LC, and HCC Patients

The allele and genotype distributions of CAT rs1001179, rs769217, and rs7943316 among the case and controls are presented in Table 3. The genotype distributions of rs1001179 (*P* = 0.684), rs7943316 (*P* = 0.263), and rs769217 (*P* = 0.052) were found to be in HWE in the control group.

**TABLE 3:**
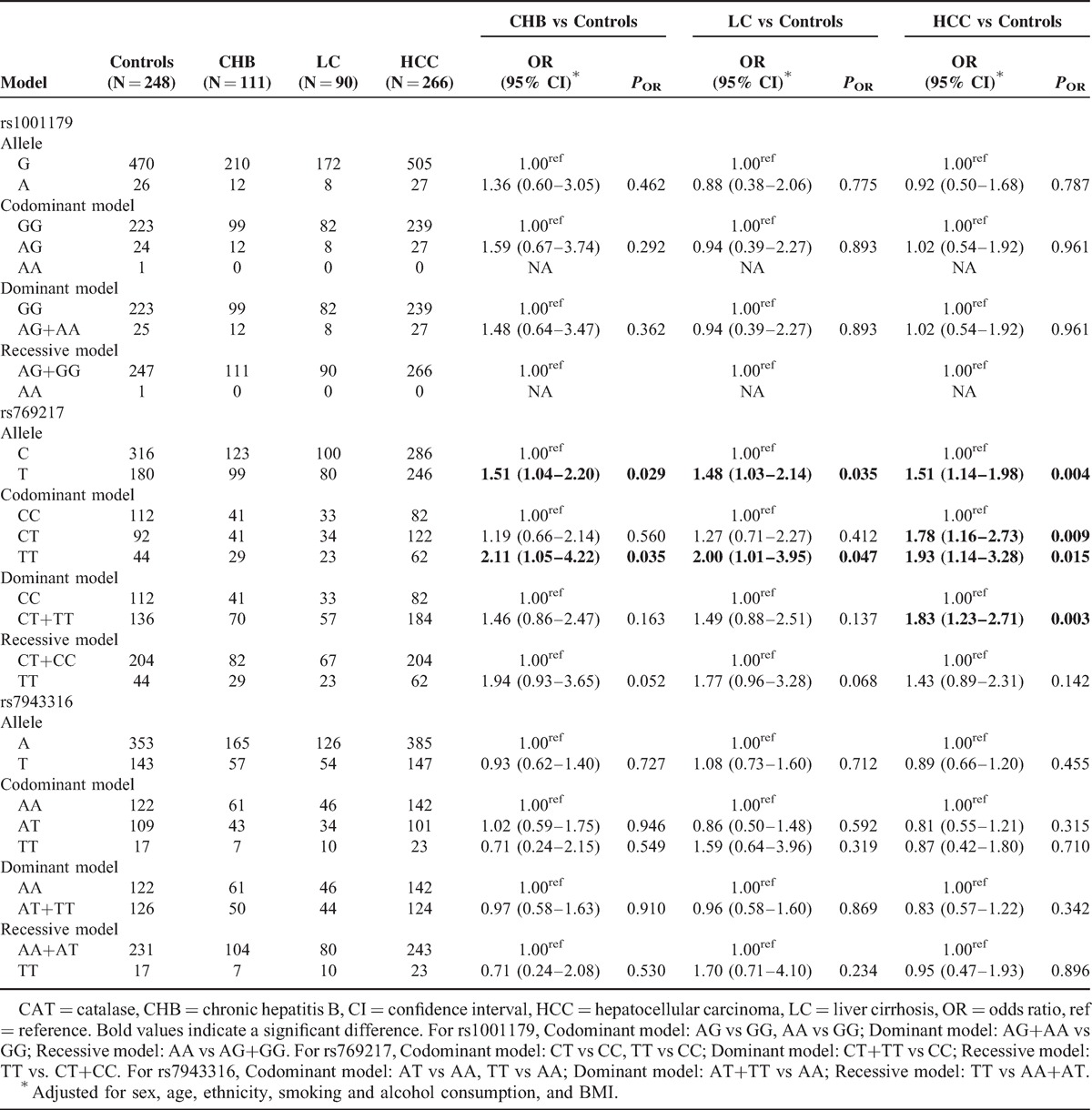
Genotype Distributions and Allele Frequencies of *CAT* Polymorphisms Between Cases and Controls

Logistic regression analysis for the CAT rs1001179 polymorphism (after adjusting for sex, age, ethnicity, smoking, alcohol consumption, and BMI) revealed no differences in allele and genotype distribution frequencies between cases and controls. The CAT rs1001179 polymorphisms were not associated with CHB, LC, and HCC risk in any analytic models. Similarly, logistic regression analyses did not reveal any significant difference in CAT rs7943316 polymorphisms between controls and cases (Table 3).

Analysis for the CAT rs769217 polymorphism indicated that subjects carrying the rs769217 T allele were at marginally increased risk of CHB, LC, and HCC when compared to carriers of the C allele, with adjusted ORs of 1.51 (95% CI = 1.04–2.20, *P* = 0.029), 1.48 (95% CI = 1.03–2.14, *P* = 0.035), and 1.51 (95% CI = 1.14–1.98, *P* = 0.004), respectively. Similarly, those individuals carrying the rs769217 TT genotype had a moderately increased risk of CHB, LC, and HCC relative to the CC genotype, with adjusted ORs of 2.11 (95% CI = 1.05–4.22, *P* = 0.035), 2.00 (95% CI = 1.01–3.95, *P* = 0.047), and 1.93 (95% CI = 1.14–3.28, *P* = 0.015), respectively. Moreover, subjects carrying the rs769217 CT genotype and at least 1 copy of the T allele (dominant model) were 1.78 times and 1.83 times more likely to develop HCC, respectively (OR = 1.78, 95% CI = 1.16–2.73, *P* = 0.009 and OR = 1.83, 95% CI = 1.23–2.71, *P* = 0.003) (Table 3).

We also developed binary logistic regression models to estimate ORs to test the association of the various genotypes and the risk of HCC compared to the LC and CHB patients. The results did not indicate any significant difference in the alleles and genotype distributions of CAT polymorphisms for HBV-related HCC risk when using CHB and LC patients as references (data not shown).

### Haplotype Distributions of CAT Polymorphisms in Healthy Controls, CHB, LC, and HCC Patients

We further performed the haplotype analysis using SHEsis software^31^ to evaluate the haplotype frequencies of polymorphisms located nearby at the same chromosome regions. The CAT rs1001179, rs769217, and rs7943316 haplotype frequencies are represented in Table 4. A total of 7 haplotypes were derived from the observed genotypes. The highest frequency of haplotype in controls was GCA, and in cases it was GAT. The frequency of the haplotype GTA was significantly associated with increased CHB (OR = 1.45, 95% CI = 1.05–2.01, *P* = 0.025) and HCC (OR = 1.49, 95% CI = 1.16–1.92, *P* = 0.002) risk. In contrast, we found 1 protective haplotype for HCC risk: GCA (OR = 0.67, 95% CI = 0.52–0.87, *P* = 0.003).

**TABLE 4:**
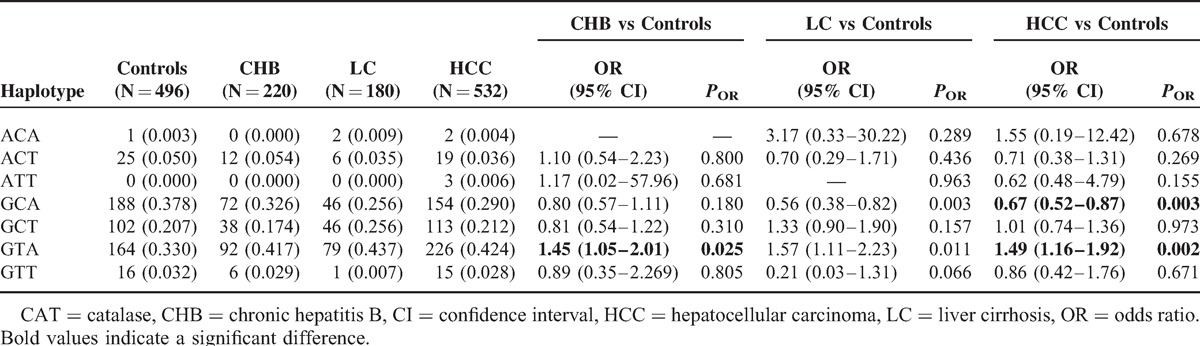
Analysis of *CAT* Haplotype Frequencies With the Risk of CHB, LC, and HCC

### Genotype Distributions of CAT Polymorphisms According to Sex, Age, Smoking, and Alcohol Consumption in Healthy Controls and Patients With HCC

To investigate the effect of other potential confounding variables on the association between *CAT* genetic variants and HCC risk, we stratified our population according to sex, age, history of smoking, and alcohol consumption.

When we stratified our population by sex, we found that male subjects carrying the rs769217 CT, TT, and the combined CT+TT genotypes were at increased risk of HCC (CT vs CC genotype: OR = 1.78, 95% CI = 1.10–2.89, *P* = 0.019; TT vs CC genotype: OR = 2.05, 95% CI = 1.12–3.76, *P* = 0.020; dominant model: OR = 1.85, 95% CI = 1.19–2.89, *P* = 0.007). No differences were observed in the genotype distributions of CAT rs1001179 and rs7943316 or in female subjects (see Table 1, Supplemental Content, http://links.lww.com/MD/A235, which illustrates the genotype distributions of CAT polymorphisms estimated by sex).

The stratification of our population by age indicated that younger (<50 years) subjects carrying the rs769217 TT genotype and at least 1 copy of the T allele (dominant model) had a significantly increased risk of HCC relative to the CC genotype, with adjusted ORs of 2.19 (95% CI = 1.05–4.58) and 1.88 (95% CI = 1.08–3.28), respectively (see Table 2, Supplemental Content, http://links.lww.com/MD/A235, which illustrates the genotype distributions of CAT polymorphisms estimated by age).

Next, we investigated the effect of tobacco smoking on the association between CAT polymorphism and HCC risk. Patients with no history of smoking and with the rs769217 CT genotype and at least 1 copy of the T allele (dominant model) were 1.79 times and 1.76 times more likely to develop HCC compared with those carrying the CC genotype (OR = 1.79, 95% CI = 1.06–3.03, *P* = 0.030 and OR = 1.76, 95% CI = 1.09–2.84, *P* = 0.020) (see Table 3, Supplemental Content, http://links.lww.com/MD/A235, which illustrates the genotype distributions of CAT polymorphisms estimated by tobacco smoking).

The stratification related to alcohol consumption indicated that patients with no history of alcohol consumption and who carried the rs769217 CT, TT, and the combined CT+TT genotypes had increased risk of HCC (CT vs CC genotype: OR = 1.85, 95% CI = 1.10–3.13, *P* = 0.021; TT vs CC genotype: OR = 1.92, 95% CI = 1.02–3.62, *P* = 0.044; dominant model: OR = 1.87, 95% CI = 1.16–3.03, *P* = 0.010) (see Table 4, Supplemental Content, http://links.lww.com/MD/A235, which illustrates the genotype distributions of CAT polymorphisms estimated by alcohol consumption).

## DISCUSSION

The current study assessed the influence of 3 common SNPs polymorphisms in the *CAT* gene on CHB, HBV-LC, and HBV-HCC risk in 715 Chinese subjects. Our results revealed a statistically significant association between CAT rs769217 polymorphisms and CHB, LC, and HCC risk. We found that the CAT rs769217 T allele and TT genotype were both significantly associated with increased CHB, LC, and HCC risk. In addition, the heterozygous rs769217 CT genotype and dominant model (combined CT and TT genotypes) were correlated with a significant increased HCC risk when compared with the CC homozygote. Moreover, we found 1 high-risk haplotype (GTA) for CHB (OR = 1.45, 95% CI = 1.05–2.01) and HCC (OR = 1.49, 95% CI = 1.16–1.92) and 1 protective haplotype (GCA) for HCC (OR = 0.67, 95% CI = 0.52–0.87). The stratification analysis by different potential confounding variables demonstrated that the CAT rs769217 T allele enhances the HCC risk among men, younger patients, patients who do not smoke, and patients who do not consume alcohol. No significant difference was found in the distribution of genotypes and allele frequencies between cases and healthy controls for CAT rs1001179 and rs7943316 polymorphisms.

Associations between oxidative stress and hepatocarcinogenesis have generally been investigated.^6,7^ CAT, which is an endogenous antioxidant enzyme, can catalyze H_2_O_2_ to O_2_ and H_2_O, thus preventing cell injury from ROS.^10^ Therefore, CAT plays a significant role in protecting cells against severe oxidative stress. The functions of CAT derive from the polymorphisms of the *CAT* gene. Genetic variations in the CAT enzyme may modulate disease risk.^11^ Several studies have suggested that CAT polymorphisms might be associated with a risk of various cancers such as breast cancer,^20^ cervical cancer,^21^ prostate cancer,^22^ pancreatic cancer,^23^ and colorectal cancer.^24^

Until now, there were only 3 studies that investigated the association between CAT polymorphisms and HCC risk.^25,26^ The first association between CAT polymorphisms and HCC risk was reported by Lee et al in 2002.^27^ They included 106 patients with HCC, but found no associations between *CAT* gene rs7943316 polymorphism and HCC risk. In 2009, Nahon et al^25^ assessed 190 HCC patients with alcoholic cirrhosis but also found no associations between *CAT* gene rs1001179 polymorphisms and HCC risk. Another study was conducted by Ezzikouri et al^26^ in 2010 and involved 96 Moroccan patients with HCC. In contrast, Ezzikouri et al^26^ reported that male patients carrying CAT rs1001179 (−262 C > T) of the TT genotype had a significantly higher risk of developing HCC when compared with controls (OR = 15.94, 95% CI = 3.48–72.92, *P* < 0.001). However, these studies were both conducted with small sample sizes and investigated only 1 SNP of the *CAT* gene. Our present study investigated the association between 3 SNPs (rs1001179, rs769217, and rs7943316), polymorphisms of the *CAT* gene, and HBV-HCC risk in a larger sample size (111 CHB patients, 90 LC, 266 HCC, and 248 controls). Our findings suggested a significant association between CAT rs769217 polymorphisms and HCC development risk. With a larger total number of subjects, more robust results were obtained in the present study than in previous studies.

Our results suggest a gender effect of the CAT rs769217 polymorphism on HCC risk. Men with the T allele had a significantly increased risk for HCC (CT vs CC genotype: OR = 1.78, 95% CI = 1.10–2.89, *P* = 0.019; TT vs CC genotype: OR = 2.05, 95% CI = 1.12–3.76, *P* = 0.020; dominant model: OR = 1.85, 95% CI = 1.19–2.89, *P* = 0.007). No differences were observed in the genotype distributions of CAT rs769217 in female subjects. The evidence for an increased risk of HCC in women but not in men is suggestive but not conclusive. The mechanisms underlying this gender difference are also still unknown. However, evidence has suggested that men exhibit lower CAT enzyme activity, suggesting that the male subgroup may be especially susceptible to cancer.^32^ On the other hand, the gender differences may be due to different dietary habits (such as consumption of fruit and vegetables) and differences in smoking and alcohol consumption between male and female patients.^33^

In our study, the CAT rs769217 TT genotype was significantly associated with increased HCC risk among subjects <50 years (OR = 2.19, 95% CI = 1.05–4.58). In 2005, Fulle et al^34^ have demonstrated that CAT enzymatic activity is significant higher in older than in younger individuals. Low CAT activity resulting from genetic variations in the CAT enzyme could alter ROS detoxification and increase oxidative stress, implicating oxidative DNA damage and thus increased risk of cancer among younger individuals.^11^

The current studyalso indicated that the association between the CAT rs769217 T allele polymorphisms and HCC risk varied according to patient history of smoking and alcohol consumption. The results unexpectedly suggested that nonsmokers and nondrinkers with rs769217 T allele are more likely to develop HCC. The evidence for these differences is suggestive but not conclusive. One of the potential explanations may be the result of the limited numbers. After stratifying our population by smoking and drinking status, sample size should be smaller in each subgroup. From our data, for rs769217, the OR values are similar between smokers and nonsmokers, drinkers and nondrinkers, although the *P* values for the smokers and drinkers are not significant. This may be due to the limitation of numbers. Thus, the results would lack statistical power and robustness, and should be read with caution. However, we could not exclude the possibility that the difference was due to smoking and drinking status. The other possible reasons for these effects may be as follows: data from previous studies indicated that heavy smokers tend to have increased local CAT activities compared to light or nonsmokers.^35^ There was also evidence indicating that CAT activity can be degraded by ethanol administration; thus, CAT may have only a limited role in inactivating H_2_O_2_ in alcoholics.^36^ Therefore, a great confusion has arisen regarding the smoking and drinking status difference in the association between the CAT rs769217 polymorphisms and HCC risk, and these findings need to be confirmed by larger studies further.

Several potential limitations of this study must be acknowledged. First, subjects in our study were recruited from only 1 hospital and limited to the Guangxi population. They may not be well representative of the entire Chinese population. Therefore, these findings may not be generalized to other populations. Second, the current research studied only 3 SNPs in the *CAT* gene. It would be interesting to identify more SNPs in the *CAT* gene to investigate their associations with HCC risk. Thus, the results of this research must be interpreted cautiously considering objectives and limitations of this study.

In conclusion, our findings indicate that the CAT rs769217 T allele has a significant association with increased risk of CHB, HBV-LC, and HBV-HCC in the Guangxi Chinese population.

## Acknowledgment

The authors would like to thank Scribendi.com for its linguistic assistance during the preparation of this manuscript.
